# Comparison of the therapeutic effects of human and mouse adipose-derived stem cells in a murine model of lipopolysaccharide-induced acute lung injury

**DOI:** 10.1186/scrt161

**Published:** 2013-01-29

**Authors:** Shijia Zhang, Svitlana D Danchuk, Kathleen MP Imhof, Julie A Semon, Brittni A Scruggs, Ryan W Bonvillain, Amy L Strong, Jeffrey M Gimble, Aline M Betancourt, Deborah E Sullivan, Bruce A Bunnell

**Affiliations:** 1 Center for Stem Cell Research and Regenerative Medicine, School of Medicine, Tulane University, 1430 Tulane Avenue, SL-99, New Orleans, LA 70112, USA; 2 Department of Pharmacology, School of Medicine, Tulane University, 1430 Tulane Avenue, SL-83, New Orleans, LA 70112, USA; 3 Department of Cell and Molecular Biology, School of Science and Engineering, Tulane University, 6400 Freret Street, New Orleans, LA 70118, USA; 4 Stem Cell Biology Laboratory, Pennington Biomedical Research Center, Louisiana State University System, 6400 Perkins Road, Baton Rouge, LA 70808, USA; 5 Department of Microbiology and Immunology, School of Medicine, Tulane University, 1430 Tulane Avenue, SL-38, New Orleans, LA 70112, USA; 6 Division of Regenerative Medicine, Tulane National Primate Research Center, 18703 Three Rivers Road, Covington, LA 70433, USA

## Abstract

**Introduction:**

Adipose-derived stem cells (ASCs) have emerged as important regulators of inflammatory/immune responses *in vitro *and *in vivo *and represent attractive candidates for cell-based therapies for diseases that involve excessive inflammation. Acute lung injury (ALI) is an inflammatory condition for which treatment is mainly supportive due to lack of effective therapies. In this study, the therapeutic effects of ASC-based therapy were assessed *in vivo *by comparison of the anti-inflammatory properties of both human and murine ASCs in a mouse model of lipopolysaccharide (LPS)-induced ALI.

**Methods:**

Human ASCs (hASCs) or mouse ASCs (mASCs) were delivered to C57Bl/6 mice (7.5 × 10^5 ^total cells/mouse) by oropharyngeal aspiration (OA) four hours after the animals were challenged with lipopolysaccharide (15 mg/kg). Mice were sacrificed 24 and 72 hours after LPS exposure, and lung histology examined for evaluation of inflammation and injury. Bronchoalveolar lavage fluid (BALF) was analyzed to determine total and differential cell counts, total protein and albumin concentrations, and myeloperoxidase (MPO) activity. Cytokine expression in the injured lungs was measured at the steady-state mRNA levels and protein levels for assessment of the degree of lung inflammation.

**Results:**

Both human and mouse ASC treatments provided protective anti-inflammatory responses. There were decreased levels of leukocyte (for example neutrophil) migration into the alveoli, total protein and albumin concentrations in BALF, and MPO activity after the induction of ALI following both therapies. Additionally, cell therapy with both cell types effectively suppressed the expression of proinflammatory cytokines and increased the anti-inflammatory cytokine interleukin 10 (IL-10). Overall, the syngeneic mASC therapy had a more potent therapeutic effect than the xenogeneic hASC therapy in this model.

**Conclusions:**

Treatment with hASCs or mASCs significantly attenuated LPS-induced acute lung injury in mice. These results suggest a potential benefit for using an ASC-based therapy to treat clinical ALI and may possibly prevent the development of acute respiratory distress syndrome (ARDS).

## Introduction

Acute lung injury (ALI) is a common clinical occurrence that results from a number of localized and systemic pathological conditions including sepsis, trauma, shock, pneumonia, gastric aspiration, toxic ingestion, and pancreatitis. ALI can progress into a life-threatening condition known as acute respiratory distress syndrome (ARDS) particularly in critically ill patients. ALI/ARDS is characterized by acute onset of overwhelming pulmonary inflammation, bilateral infiltrates, and diffuse alveolar damage. Inflammation may progress to the point of widespread pulmonary edema and poor lung compliance that ultimately result in severe hypoxemia and devastating respiratory failure [1-3]. Studies indicate that the age-adjusted incidence of ALI/ARDS in the United States is 86.2 per 100,000 person-years, and the mortality rate is 36 to 44% due to lack of an effective therapy [4,5]. Current treatment for ALI/ARDS is mainly limited to supportive care including mechanical ventilation with concomitant treatment of underlying diseases or initiating factors [2]. Given the severe complications and high mortality rate of ALI/ARDS, a novel and more effective therapy is needed.

Multipotent stromal cells (or mesenchymal stem cells, MSCs) are a heterogeneous subset of progenitor cells that have self-renewal and multilineage differentiation capabilities [6-8]. In recent years, bone marrow-derived MSCs (BMSCs) have emerged as promising cell-based therapeutic agents for various inflammatory diseases given their more recently recognized property of potent immunomodulation and immunosuppression [8-12].

Encouraging results have been obtained using BMSC therapy in both rodent and human tissue models of ALI/ARDS [13-15]. Gupta *et al*. reported that intrabronchial delivery of murine BMSCs improved survival and attenuated lipopolysaccharide (LPS)-induced ALI in mice. This study showed that bronchoalveolar lavage fluid (BALF) and plasma from endotoxin-challenged mice treated with BMSCs had lower concentrations of tumor necrosis factor alpha (TNF-α) and macrophage inflammatory protein 2 (MIP-2) and higher concentrations of interleukin 10 (IL-10) compared with untreated mice [13]. Recently, our group reported that, in a mouse model of LPS-induced acute lung injury, administration of human BMSCs by oropharyngeal aspiration (OA) significantly reduced the expression of proinflammatory cytokines, neutrophil counts, total BALF protein and pulmonary edema. Our study demonstrated that xenogeneic BMSCs recapitulated the observed immunomodulatory effects of syngeneic BMSCs [14]. However, a critical hindrance in clinical translation of BMSCs is that bone marrow has a high degree of viral exposure [16,17], and it may not be practical to obtain BMSCs from some morbid donors [18,19].

In contrast, adipose tissue is abundant, expendable, and easily accessible, and adipose-derived MSCs (that is adipose-derived stem cells (ASCs) or adipose stem cells) can be isolated from adipose tissue using a simple protocol that results in high yields, due in part to the higher frequency of mesenchymal stem cells in the adipose tissue compared to bone marrow. Because they are easy to acquire and exhibit some of the same immunosuppressive and immunomodulatory characteristics as BMSCs [20], ASCs are an attractive alternative source of readily available adult stem cells. However, very little is known regarding the effect of adipose-derived stem cells in experimental models of ALI/ARDS. The current study tests the ability of ASCs isolated from either syngeneic mice or humans to ameliorate lung injury and suppress inflammation after administration of LPS via an OA route. This may be the first report describing the therapeutic role of mouse syngeneic ASC or human xenogeneic ASC in attenuating ALI in a murine model of LPS-induced ALI.

## Materials and methods

### Preparations of human and mouse ASCs

Human and mouse ASCs were isolated and characterized using standard procedures developed at the Pennington Biomedical Research Center [21-23]. All protocols were reviewed and approved by the Pennington Biomedical Research Center Institutional Review Board and all human participants provided written informed consent (PBRC number 23040). The mASCs were isolated from inbred transgenic C57Bl/6-Tg(UBC-GFP)30Scha/J mice (Jackson Laboratories, Bar Harbor, ME, USA), which ubiquitously express enhanced green fluorescent protein (eGFP) in all tissues. The cells were negative for CD11b, CD31, and CD45, and they were positive for CD29 and CD106 via flow cytometry analysis (data not shown). The cells were able to generate colony-forming units-fibroblasts (CFU-Fs, data not shown) and were able to differentiate along adipogenic and osteogenic lineages (data not shown). Frozen vials of 1 × 10^6 ^human or mouse ASCs (passage 3) were thawed and plated onto a 145 cm^2 ^culture dish (Nunc, Rochester, NY, USA) in 20 ml complete culture medium (CCM) that consisted of DMEM/F-12 (GIBCO, Grand Island, NY, USA), 10% fetal bovine serum (Atlanta Biologicals, Lawrenceville, GA, USA), and 1% Anti-Anti (antibiotic-antimycotic, GIBCO). The cells were incubated at 37°C with humidified air containing 5% CO_2_. After 18 to 20 hours, the medium was removed and adherent cells were washed once with 1 × phosphate-buffered saline (PBS, GIBCO), harvested with 0.25% trypsin/1mM EDTA (GIBCO), and replated at 200 cells/cm^2 ^in CCM for expansion. Media were changed every 3 to 4 days until the cells reached approximately 70% confluence. The culture medium was changed to antibiotic-antimycotic-free CCM one day before the cells were to be delivered to mice. ASCs were harvested with 0.25% trypsin/1 mM EDTA, washed with PBS, resuspended in Hank's balanced salt solution (HBSS, GIBCO) and administered to mice via OA as described below. HBSS alone was used as a vehicle-only control.

### ALI induction and treatment protocols

All animal procedures were approved by the Institutional Animal Care and Use Committee (IACUC) at Tulane University and conformed to the requirements of the Animal Welfare Act.

To induce acute lung injury, female C57Bl/6 mice (National Cancer Institute-Frederick, Frederick, MD, USA) aged 8 to 10 weeks were first anesthetized by inhalation of 2% isoflurane (VetOne, Meridian, ID, USA) vapor mixed with oxygen as previously described [14,24]. Anesthetized mice were suspended by their cranial incisors, and the tongue was extracted to full extension to prevent the swallowing reflex. LPS from *Escherichia coli *055:B5 (15 mg/kg, Sigma-Aldrich, St. Louis, MO, USA) or PBS vehicle alone (75 µl each) was pipetted into the back of the throat, and the nares were pinched shut to force breathing through its mouth and liquid aspiration. Four hours after LPS challenge, two doses of human or mouse ASCs (3.75 × 10^5^, suspended in 75 µl HBSS) or HBSS vehicle-only control (75 µl each) were delivered by OA 30 minutes apart, for a total dose of 7.5 × 10^5 ^cells (150 µl total volume) per mouse for each test group. The animals were sacrificed 24 or 72 hours after LPS exposure by exsanguination under anesthesia with 80 mg/kg ketamine plus 8 mg/kg xylazine. The lungs were processed for bronchoalveolar lavage (BAL) collection, RNA or protein isolation, or histology as described below.

### Bronchoalveolar lavage

Immediately after exsanguination, the lungs were cannulated with a 20-gauge intravenous (IV) catheter (Exel International Medical Products, St. Petersburg, FL, USA) and gently washed five times with 530 μl (right lung) or 1 ml (whole lung) PBS supplemented with 0.4 mM EDTA (GIBCO) and protease inhibitor cocktail (Roche, Indianapolis, IN, USA). The lavage fluid was spun at 1,500 × g for 5 minutes at 4°C to pellet the cells. Cells from all five lavage collections were pooled for total cell counting while the supernatant from the first lavage was stored at-80°C for biochemical analysis. The protein concentration in the bronchoalveolar lavage fluid was measured using the micro bicinchoninic acid (BCA) assay kit (Pierce, Rockford, IL, USA). For differential cell counts, the cells were spun onto glass slides by cytospin (Thermo-Shandon, Wilmington, DE, USA) and stained with a modified Wright-Giemsa stain (Diff-Quik, Fisher Scientific, Pittsburgh, PA, USA). The numbers of neutrophils, macrophages, eosinophils, basophils, and lymphocytes, up to a total of 100 cells, were determined in three random viewing fields per sample.

### Hematoxylin and eosin (H&E) staining

Lungs were inflation-fixed with 10% neutral buffered formalin (Sigma-Aldrich) through the trachea at 25 cm H_2_O pressure for 20 minutes, excised from the mice, and placed in fresh 10% neutral buffered formalin at 4°C. The fixed lungs were then embedded in paraffin, and 6 μm sections were stained with H&E to reveal anatomic details. Whole section images were captured on an Aperio ScanScope (Aperio Technologies, Vista, CA, USA) and viewed using Aperio ImageScope (Aperio Technologies).

### Lung homogenization, RNA isolation, and cDNA synthesis

Excised lung tissue was weighed and homogenized using a Bio-Plex cell lysis kit (Bio-Rad, Hercules, CA, USA) as per the manufacturer's instruction. The homogenates were centrifuged at 10,000 × g for 15 minutes, and the supernatant was aliquoted and stored at-80°C until analyzed. The protein concentration in the supernatant was measured by BCA assay. Total RNA was isolated from lung tissue homogenized in TriPure Isolation Reagent (Roche) and was purified with the RNeasy mini kit (Qiagen, Valencia, CA, USA). The RNA was first treated with DNase I (Amplification grade, Invitrogen) and then converted into cDNA using iScript cDNA Synthesis Kit (Bio-Rad) following the manufacturer's instructions in a PTC-200 Peltier Thermal Cycler (MJ Research, Ramsey, MN, USA).

### Real-time RT-PCR

The persistence of viable hASCs or mASCs in the lung was determined at 20 hours after the cells were delivered using real-time RT-PCR for human glyceraldehyde-3-phosphate dehydrogenase (GAPDH) or eGFP mRNA, respectively, as previously described [14,25]. TaqMan Gene Expression Assay (Hs00266705_g1, Applied Biosystems, Foster City, CA, USA) was used to specifically quantify human GAPDH. The following primer/probe set was used to quantify eGFP: forward 5'-CCACATGAAGCAGCAGGACTT-3', reverse 5'-GGTGCGCTCCTGGACGTA-3', and probe 5'-6FAM-TTCAAGTCCGCCATGCCCGAA-TAMRA-3'. Briefly, 7.5 × 10^5 ^hASCs or mASCs were added to a whole lung tissue sample just prior to homogenization and RNA isolation. Standard curves were generated by adding serial dilutions of mRNA isolated from lung tissue plus ASCs to mRNA isolated only from lung tissue. Real-time RT-PCR was performed, and Ct values were used to determine the persistence of hASCs or mASCs according to the standard curves. The final value for total cDNA in the sample was corrected by parallel real-time RT-PCR assays with primers that amplified both the human and the mouse genes for GAPDH (forward 5'-CAGCGACACCCACTCCTCCACCTT-3', reverse 5'-CATGAGGTCCACCACCCTGTTGCT-3') and mouse ribosomal protein, large, P0 (Rplp0, forward 5'-CGACCTGGAAGTCCAACTAC-3', reverse 5'-ATCTGCTGCATCTGCTTG-3'). Results were presented as the percentage of the calculated cell number relative to the delivered cell number (7.5 × 10^5^). For cytokine/chemokine expression analysis, the real-time PCR reactions were performed as previously described by Ripoll *et al*. with modifications [9]. Briefly, each reaction mixture contained 1 µl of commercially available TaqMan GeneExpression Assay primer/probe set (Applied Biosystems), 10 µl TaqMan Gene Expression Master Mix (Applied Biosystems), 50 ng cDNA template and RNase-free water with a total volume of 20 µl. Beta-actin (β-actin) was used as an endogenous control. The reaction was performed at 50°C for 2 minutes, 95°C for 10 minutes followed by a 40-cycle two-step PCR (95°C for 15 seconds and 60°C for 1 minute) using the CFX96 Real-Time System (Bio-Rad). TaqMan GeneExpression Assays (Applied Biosystems) used for murine genes were: β-actin (Mm00607939_s1), macrophage inflammatory protein 2 (MIP-2, Mm00436450_m1), interleukin 1 alpha (IL-1α, Mm00439621_m1), IL-1β (Mm01336189_m1), MIP-1α (Mm00441258_m1), and tumor necrosis factor alpha (TNF-α, Mm00443258_m1), and IL-10 (Mm01288386_m1).

### Myeloperoxidase activity assay, ELISA, and multiplex immunoassay

BALF myeloperoxidase (MPO) activity assay was performed as previously described [14]. Briefly, BALF was added to a reaction buffer containing 50 mM potassium phosphate (pH 6.0), 0.0005% (v/v) H_2_O_2_, and 0.167 mg/ml o-dianisidine dihydrochloride (Sigma-Aldrich); the absorbance at 460 nm was monitored for 30 minutes using the Synergy HT multi-detection microplate reader (Bio-Tek Instruments, Winooski, VT, USA). The albumin levels in BALF were measured using a mouse albumin ELISA kit purchased from Bethyl Laboratories (Montgomery, TX, USA). Measurement of cytokines and chemokines in lung homogenates was performed by multiplex immunoassay using a Millipore mouse cytokine/chemokine 32-plex kit (Millipore, Billerica, MA, USA).

### Statistical analysis

Data are summarized as mean ± SEM. One-way ANOVA was performed for statistical analysis, followed by Bonferroni multiple comparison tests. The statistical significance value was set at *P *<0.05.

## Results

### Engraftment of hASCs and mASCs in the lung

**Figure 1:**
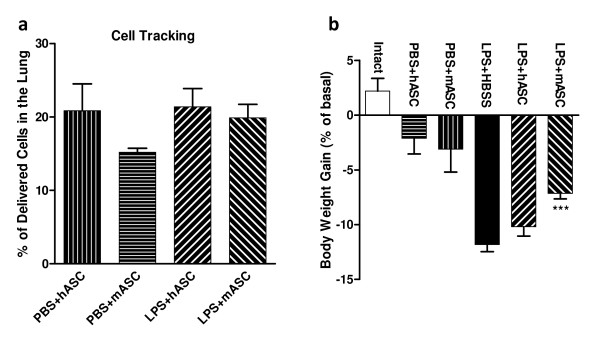
**Persistence of hASCs and mASCs and change in body weight**. **(a) **The percentages of total injected hASCs or mASCs present in the lung 20 hours after oropharyngeal administration for PBS-and LPS-challenged mice. Real-time RT-PCR analysis using human GADPH and eGFP primers was used to track and quantify the hASCs and mASCs, respectively. N = 4, 4, 4, and 5 for PBS + hASC, PBS + mASC, LPS + hASC, and LPS + mASC, respectively. **(b) **Changes in body weight following treatment with HBSS, hASC, or mASC were compared 24 hours after PBS or LPS challenge. N = 7, 6, 6, 16, 10, and 10 for Intact, PBS + hASC, PBS + mASC, LPS + HBSS, LPS + hASC, and LPS + mASC, respectively. Significance was defined as *** for *P *<0.001, for comparison of the experimental cell treatment groups to the HBSS-treated LPS-challenged mice. eGFP, enhanced green fluorescent protein; GAPDH, glyceraldehyde-3-phosphate dehydrogenase; hASCs, human adipose-derived stem cells; HBSS, Hank's balanced salt solution; LPS, lipopolysaccharide; mASCs, mouse adipose-derived stem cells; PBS, phosphate-buffered saline; RT-PCR, reverse transcription-polymerase chain reaction.

Real-time RT-PCR for human GAPDH or eGFP was performed to track hASCs and mASCs, respectively, in the lungs 20 hours after being delivered through oropharyngeal aspiration. It was determined that 20.85 ± 3.65% (N = 4) of administered hASCs could be found in the lungs in the PBS + hASC group, whereas there was 21.38 ± 2.49% (N = 4) in the LPS + hASC group based on real-time RT-PCR results. For mASCs, 15.18 ± 0.55% (N = 4) of delivered cells were detected in the PBS + mASC group, while 19.88 ± 1.83% (N = 5) in the LPS + mASC group (Figure 1a).

### Change in body weight following treatment

The body weight of each mouse was determined immediately prior to LPS (or PBS control) challenge and again at the time of sacrifice (24 hours after LPS exposure). The change in body weight was calculated as percentage relative to the basal body weight. While intact, untreated mice (N = 7) gained about 2% body weight during this 24-hour period, mice in the PBS + hASC (N = 6) and PBS + mASC (N = 6) control groups lost 2 to 3% body weight during the 24-hour period. For the diseased mice induced by LPS, the percentage of body weight lost was reduced following hASC (10.19 ± 0.85%, N = 10, *P *>0.05) and mASC (7.14 ± 0.52%, N = 10, *P *<0.001) treatment, as compared to the HBSS vehicle-only controls (11.82 ± 0.66%, N = 16) (Figure 1b).

### Assessment of lung histology and microvascular permeability

**Figure 2:**
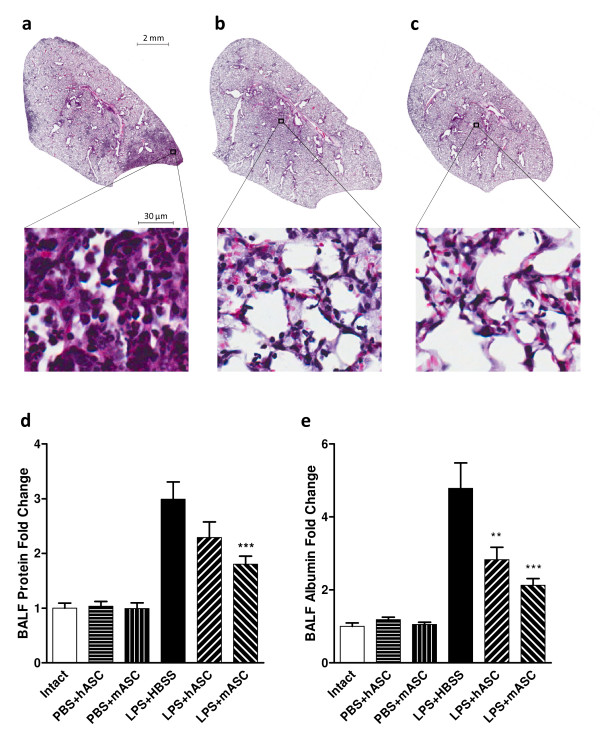
**hASCs and mASCs improved severity of lung injury**. Representative H&E lung sections 72 hours following LPS challenge for mice treated with **(a) **HBSS (N = 5), **(b) **hASCs (N = 5), or **(c) **mASCs (N = 5). Comparisons at the alveolar level (enlarged images) show decreased cellularity, septal thickening, and exudation when hASCs or mASCs were administered following LPS challenge. Levels of **(d) **protein and **(e) **albumin in the bronchoalveolar lavage fluid were used to assess the extent of vascular leakage after HBSS or ASC treatment. Control groups with intact or PBS-challenged mice were included to assess any changes in these levels due to the injection method or cell treatment. Values were presented as fold changes relative to intact, untreated mice. For (d) and (e), N = 3, 6, 6, 10, 9, and 10 for Intact, PBS + hASC, PBS + mASC, LPS + HBSS, LPS + hASC, and LPS + mASC, respectively. Significance was defined as ** and *** for *P *<0.01 and *P *<0.001, respectively, for comparison of the experimental cell treatment groups to the HBSS-treated LPS-challenged mice. ASC, adipose-derived stem cell; hASCs, human adipose-derived stem cells; HBSS, Hank's balanced salt solution; H&E, hematoxylin and eosin; LPS, lipopolysaccharide; mASCs, mouse adipose-derived stem cells; PBS, phosphate-buffered saline.

To determine the effect of hASCs or mASCs treatment on the lung injury, H&E staining of lung sections from animals 72 hours after LPS challenge was examined using an Aperio ScanScope. Both hASCs and mASCs markedly decreased lung injury after LPS exposure; specifically, both cell types reduced cellularity, exudation and septal thickening compared to HBSS vehicle-only controls (Figure 2a-c).

Massive inflammation causes vascular leakage in the lung in this LPS-induced ALI mouse model [13,14], as indicated by the elevated levels of protein and albumin in BALF (Figure 2d, e). To assess the effect of hASCs or mASCs on the integrity of the alveolar-capillary membrane barrier and pulmonary vascular leakage, the levels of total protein and albumin in the bronchoalveolar lavage fluid were examined 24 hours after PBS or LPS challenge. Results demonstrated that total protein concentration in BALF was reduced in both the hASC (N = 9) and mASC (N = 10) treatment groups compared to the HBSS-only control group (N = 10), however, statistical significance (*P *<0.001) was reached only in the mASC treatment group. Administration of hASCs (N = 9) and mASCs (N = 10) resulted in significantly lower concentrations of BALF albumin, about 41% and about 56% lower than those of HBSS (N = 10) vehicle-treated mice (*P *<0.01 and *P *<0.001, respectively). Interestingly, these parameters showed a trend that treatment with mASCs decreased BALF total protein and albumin even more than hASC treatment, although this effect was not statistically significant.

### Effect of hASCs and mASCs on the infiltration of inflammatory cells

**Figure 3:**
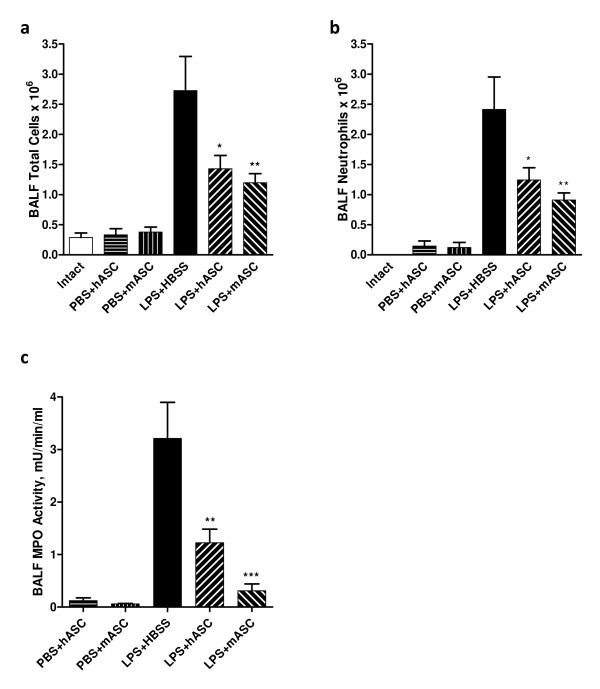
**Characterization of human and mouse ASC effects on inflammatory cell infiltration in the **lung **after LPS injury**. Administration of hASCs or mASCs, when compared to HBSS, significantly decreased the **(a) **total cell number, **(b) **infiltrating neutrophil number as determined by a modified Wright-Giemsa stain, and **(c) **MPO activity in BALF 24 hours after LPS exposure. For (a) and (b), N = 3, 6, 6, 10, 9, and 10 for Intact, PBS + hASC, PBS + mASC, LPS + HBSS, LPS + hASC, and LPS + mASC, respectively. For (c), N = 4, 3, 5, 4, and 5 for PBS + hASC, PBS + mASC, LPS + HBSS, LPS + hASC, and LPS + mASC, respectively. Significance was defined as *, **, and *** for *P *<0.05, *P *<0.01, and *P *<0.001, respectively, as compared to the HBSS-treated LPS-challenged mice. ASC, adipose-derived stem cell; BALF, bronchoalveolar lavage fluid; hASCs, human adipose-derived stem cells; HBSS, Hank's balanced salt solution; LPS, lipopolysaccharide; mASCs, mouse adipose-derived stem cells; MPO, myeloperoxidase; PBS, phosphate-buffered saline.

Inflammatory cell migration and infiltration into the site of inflammation is extremely important in the tissue damage of lung injury [26]. To evaluate the effect of hASCs and mASCs on inflammatory cell infiltration in the lung after LPS injury, the total cell number in BALF was counted using a hemocytometer. The BAL total cell number 24 hours after LPS challenge in the HBSS control group was 2.73 × 10^6 ^± 0.57 × 10^6 ^(N = 10), while this number was significantly decreased due to the administration of hASCs or mASCs to 1.43 × 10^6 ^± 0.22 × 10^6 ^(N = 9, *P *<0.05) or 1.20 × 10^6 ^± 0.15 × 10^6 ^(N = 10, *P *<0.01), respectively (Figure 3a). A modified Wright-Giemsa stain was performed to differentiate the different cell types in BAL. The vast majority of infiltrating cells were neutrophils in all LPS injury groups. Treatment with hASC and mASC reduced the infiltrating neutrophil number from 2.41 × 10^6 ^± 0.54 × 10^6 ^(N = 10) in the control to 1.24 × 10^6 ^± 0.20 × 10^6 ^(N = 9, *P *<0.05) and 0.91 × 10^6 ^± 0.12 × 10^6 ^(N = 10, *P *<0.01), respectively (Figure 3b). MPO activity in BALF 24 hours after LPS challenge was also analyzed as an alternative measure of neutrophil infiltration in the tissue under pathological conditions. In both hASC and mASC treatment groups, MPO activity was significantly decreased from the high level of 3.21 ± 0.69 mU/min/ml (N = 10) in control mice to 1.23 ± 0.26 mU/min/ml (N = 9, *P *<0.05) and 0.31 ± 0.13 mU/min/ml (N = 10, *P *<0.01), respectively (Figure 3c). There were fewer BAL total cells and neutrophils, as well as lower MPO activity, in the mASCs group as compared to the hASCs treatment group; however, these differences were not found to be statistically significant.

### Regulation of cytokine expression by hASCs and mASCs

**Figure 4:**
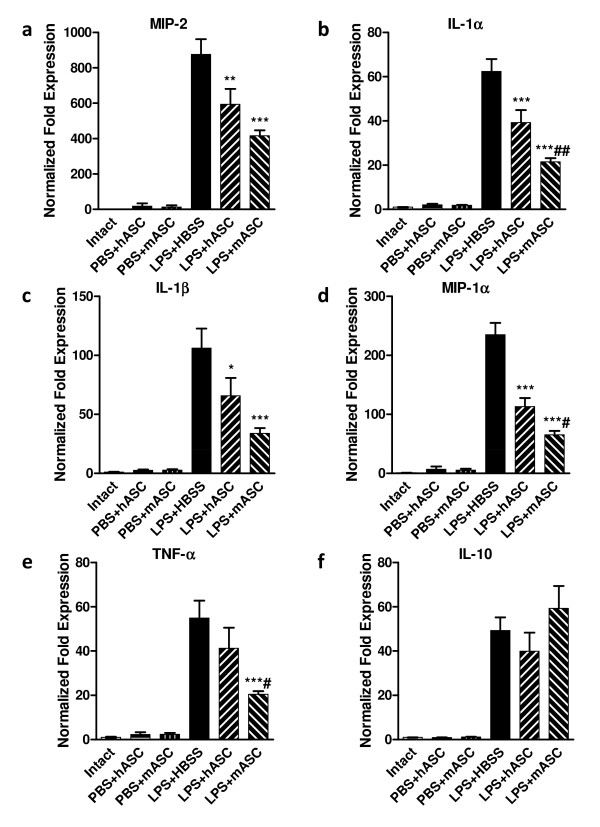
**Regulatory effects of hASCs and mASCs on inflammatory responses in the lung**. The lung mRNA levels of various proinflammatory cytokines and chemokines 24 hours after PBS or LPS challenge were quantified using real-time RT-PCR. All levels were normalized to beta-actin and reported as fold changes compared to unchallenged and untreated mouse levels. Steady-state mRNA levels of **(a) **macrophage inflammatory protein 2 (MIP-2), **(b) **interleukin 1 alpha (IL-1α), **(c) **IL-1β, **(d) **MIP-1α, **(e) **tumor necrosis factor alpha (TNF-α), and **(f**) IL-10 are shown for PBS-or LPS-challenged mice injected with HBSS, hASCs, or mASCs. N = 2, 4, 4, 5, 4, and 5 for Intact, PBS + hASC, PBS + mASC, LPS + HBSS, LPS + hASC, and LPS + mASC, respectively. Significance was defined as *, **, and *** for *P *<0.05, *P *<0.01, and *P *<0.001, respectively, compared to control groups; significant differences between the LPS-challenged cell-treatment groups (LPS + hASC and LPS + mASC) were denoted by ## for *P *<0.01. hASCs, human adipose-derived stem cells; HBSS, Hank's balanced salt solution; LPS, lipopolysaccharide; mASCs, mouse adipose-derived stem cells; PBS, phosphate-buffered saline; RT-PCR, reverse transcription-polymerase chain reaction.

To determine the regulatory effects of hASC and mASCs on inflammatory responses in the lung, total RNA was isolated from the lung tissue and the mRNA levels of various proinflammatory cytokines and chemokines were assessed by quantitative, real-time RT-PCR. ß-actin was used as an endogenous control, and the gene expression level was normalized to the unchallenged and untreated mice to show fold changes. As expected, the lungs from the PBS-challenged control mice treated with hASCs (N = 4) or mASCs (N = 4) had very low levels of the inflammatory markers, while those challenged with LPS and treated with HBSS (N = 5) increased the expression of proinflammatory cytokines and chemokines dramatically. Treatment with hASCs (N = 4) and mASCs (N = 5) suppressed the steady-state mRNA levels of MIP-2 (*P *<0.01 and *P *<0.001, respectively), IL-1α (*P *<0.001 and *P *<0.001, respectively), IL-1β (*P *<0.05 and *P *<0.001, respectively), MIP-1α (*P *<0.001 and *P *<0.001, respectively), and TNF-α (*P *>0.05 and *P *<0.001, respectively), as compared to the HBSS-only controls (Figure 4a-e) 24 hours after LPS challenge. Treatment with mASCs suppressed the expression of IL-1α, MIP-1α, and TNF-α (*P *<0.01, *P *<0.05 and *P *<0.05, respectively) more so than treatment with hASCs. For MIP-2 and IL-1β, the data showed a similar trend, but the difference between mASC and hASC treatment was not statistically significant. With mASC treatment, the level of the anti-inflammatory cytokine IL-10 was slightly increased compared to the HBSS treatment control (*P *>0.05); however, this effect was not noticed in the hASC treatment group.

**Figure 5:**
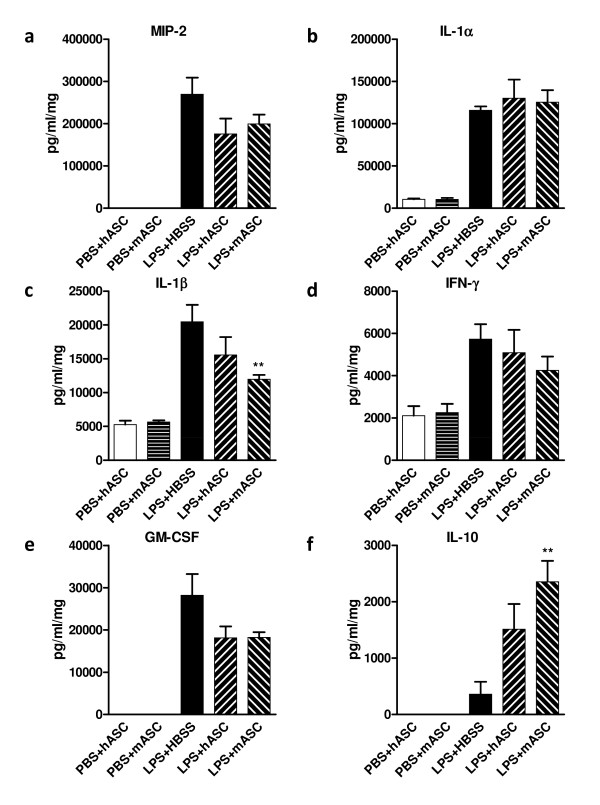
**The effects of hASC and mASCs on inflammatory cytokine levels in the lung**. Protein levels of various proinflammatory cytokines and chemokines 24 hours after PBS or LPS challenge were quantified for lung lysates using a multiplex immunoassay. All levels were normalized to total lung lysate protein concentration. Protein levels of **(a) **macrophage inflammatory protein 2 (MIP-2), **(b) **interleukin 1 alpha (IL-1α), **(c) **IL-1β, **(d) **interferon gamma (IFN-γ), **(e) **granulocyte macrophage colony-stimulating factor (GM-CSF), and **(f) **IL-10 are shown for PBS-or LPS-challenged mice injected with HBSS, hASCs, or mASCs. N = 4, 4, 5, 4, and 5 for PBS + hASC, PBS + mASC, LPS + HBSS, LPS + hASC, and LPS + mASC, respectively. Significance was defined as ** for *P *<0.01 compared to HBSS-treated LPS-challenged mice levels. hASCs, human adipose-derived stem cells; HBSS, Hank's balanced salt solution; LPS, lipopolysaccharide; mASCs, mouse adipose-derived stem cells; PBS, phosphate-buffered saline.

To further investigate the protein levels of pro-and anti-inflammatory cytokines/chemokines in the lung in response to hASCs (N = 4) or mASCs (N = 5) therapy 24 hours after LPS injury, a multiplex immunoassay was performed on mouse lung homogenates with results normalized to total protein concentration of each sample. The results showed trends for decreased production of MIP-2, IL-1β, interferon gamma (IFN-γ), and granulocyte macrophage colony-stimulating factor (GM-CSF) following hASCs and mASCs administration, without affecting the secretion of IL-1α (Figure 5a-e). The production of the anti-inflammatory cytokine IL-10 was significantly increased in the mASC treatment group (*P *<0.01) and only slightly increased in the hASC treatment group as compared to the HBSS control (N = 5).

## Discussion

According to the Berlin definition of ARDS, the term acute lung injury (ALI) has been eliminated since 2011 [27], however, the majority of the field still uses ALI as a valid denomination. For the purpose of this article, we still use the term ALI. Given that excessive inflammatory responses play a crucial role in the pathophysiology of ALI/ARDS [2], immunomodulatory therapy is an attractive new approach in the treatment of such afflictions [28,29]. Multipotent stromal cells provide unique opportunities for developing novel treatments for a large array of inflammatory diseases, because MSCs possess potent immunomodulatory properties and are often considered to be hypoimmunogenic or 'immunoprivileged' [10,30]. An emerging body of data indicates that either human or mouse bone marrow-derived MSCs (BMSCs) alleviate tissue damage and suppress inflammatory reactions in different models of ALI [13-15,31]. As an alternative source of mesenchymal stem cells, adipose tissue is abundant and easily accessible, and high yields of adipose-derived MSCs (ASCs) are easier to isolate with less invasive procedures. ASCs have been reported to have similar immune regulatory capabilities as BMSCs [20,32]. While the use of BMSCs to treat ALI/ARDS is promising, there is little known about the potential therapeutic effects of ASCs in this disease setting. In this study, the therapeutic effect of human-and mouse-derived ASCs was investigated in a mouse model of LPS-induced ALI.

Endotoxin (bacterial LPS)-induced ALI in mice is a widely used experimental model to investigate the pathogenesis and treatment of ALI/ARDS. However, it should be noted that no single ALI animal model can completely reproduce all the pathophysiological features of ALI/ARDS in humans. The airway LPS administration model employed in these studies results in massive alveolar neutrophil infiltration, which is also seen in human ALI/ARDS [33,34].

In this study the relatively noninvasive OA method [24] was used to administer both LPS and ASCs. A previous study from our group showed a comparable beneficial effect using the IV, intraperitoneal (IP), and OA methods to deliver human BMSCs to LPS-challenged mice [14].

Taking advantage of the cross-species nature of hASCs and the eGFP marker of mASCs in our experiments, the persistence of the cells in the lungs after delivery through OA was confirmed by real-time RT-PCR for human GAPDH and eGFP. It is expected that the cells detected reflect persistence of live cells since mRNA for human GAPDH and eGFP was detected as markers for human and mouse ASCs, respectively.

These results demonstrated that treatment of ALI with either hASCs or mASCs decreased the body weight loss following ALI induction, which suggests lower disease severity following treatment. Both treatments decreased LPS-induced lung damage and neutrophil infiltration substantially, as shown by H&E staining. This was further corroborated by decreased levels of total protein and albumin in BALF, indicating reduced endothelial and epithelial permeability in the lung following either hASCs or mASCs treatment.

Because neutrophil activation and transmigration into lung interstitium and broncheoalveolar space plays a crucial role in the development of ALI [26], it can be inferred that decreasing infiltration of neutrophils using ASCs administration may have clinically relevant therapeutic potential. Activated neutrophils secrete proteinases and potent enzymes such as MPO that generate oxidants, all of which can damage lung tissue leading to edema and loss of lung function [35]. Decreased MPO activity after cell treatment indicates that both hASCs and mASCs are capable of decreasing the lung inflammatory responses that ultimately cause the neutrophil-associated tissue damage common in ALI.

Nemeth *et al*. and Anderson *et al*. showed that mouse BMSCs and ASCs were able to reprogram macrophages to increase their IL-10 production, and the administration of these cells protected mice from sepsis [12,32]. In this study, both hASC and mASC treatments led to a reduction of the expression of various proinflammatory cytokines/chemokines, while increasing the production of the anti-inflammatory cytokine IL-10. These data suggest that the anti-inflammatory effects of hASC and mASC in this model were mediated, at least in part, by the increased production of IL-10 in the lung. The decrease of the production of proinflammatory cytokines/chemokines at the protein level was found to be not as pronounced as that at the mRNA level 24 hours after LPS exposure. This may be explained by the time lag between translation and transcription.

It is important to note that while cytokine and chemokine levels were quantified in the lungs of mASC-treated mice, the cellular source of the cytokines or chemokines (that is, lung epithelial cells, alveolar macrophages, inflammatory cells or administered ASCs) was not able to be determined. Our previous study showed that, when stimulated with LPS or BALF, mASCs did secrete some cytokines and chemokines (for example IL-1α and MIP-1α) *in vitro *(data not shown). These data suggest that decreased cytokine levels in animals treated with mASCs may actually be lower than recorded since the mASC may act as an exogenous source of these cytokines. However, the expression of cytokines and chemokines from human ASCs could not be detected since human cytokines and chemokines would not be detected by mouse specific primers. Therefore, using human ASCs offers the possibility of determining how the ASCs are responding to the injured lung, because human specific primers or antibodies can be used.

To the best of our knowledge, this is the first report to demonstrate the beneficial effects of ASCs for the treatment of LPS-induced acute lung injury *in vivo*. Moreover, the therapeutic effects of human and mouse ASCs have been compared side by side. While both hASCs and mASCs significantly attenuated the lung injury and inflammation, some notable differences between the two cell types were observed. For most of the parameters examined, mASCs had a more beneficial effect than xenogeneic hASCs. A possible explanation for this observation is that the cross-talk between mASCs and the injured mouse lung is more effective than in the xenotransplantation model using hASCs. In addition, studies have demonstrated species variation in the mechanisms of bone marrow-derived MSC-mediated immunosuppression. Ren *et al*. showed that under the same culture conditions, immunosuppression by human or monkey BMSCs was mediated by indoleamine 2,3-dioxygenase, while mouse BMSCs utilized nitric oxide [36]. The species variation may also be an explanation to the different therapeutic effects of hASCs and mASCs in this ALI model.

To advance the use of stem cell therapy in the treatment of ALI/ARDS it is essential that the molecular mechanisms behind any observed beneficial effects be understood. As discussed above, there is increasing evidence that both mouse and human BMSCs have similar anti-inflammatory properties, however the mechanisms mediating these outcomes may be unique to each cell type. Similarly, our unpublished data suggest that human and mouse ASCs suppress inflammation in the lung by different mechanisms. Therefore, it is essential that human cells be used in preclinical studies employing animal models. However concerns that the protective factors produced by human MSCs may not be effective in other species have limited such studies. Results from this study demonstrate potent anti-inflammatory effects of both human and mouse ASCs in wild-type mice exposed to LPS, a well-characterized model of ALI. While syngeneic mouse ASCs were expectedly more effective at suppressing inflammation, our results clearly demonstrate the feasibility of using immunocompetent mice to interrogate the molecular mechanisms by which human ASCs suppress inflammation.

There are currently more than 60 clinical trials registered with clinicaltrial.gov testing ASCs in a variety of disorders, including Crohn's fistula, type 2 diabetes, and chronic obstructive pulmonary disease. As discussed above, ASCs are easy to isolate and expand *in vitro*, and they are also 'immunoprivileged' [37], which makes allogeneic transplantation to humans feasible. Despite the promising results we obtained in the mouse model, clinicians should use caution when considering the use of ASCs as a therapy for ALI/ARDS in human subjects. A recent study showed that in both humans and mice, intravenous infusion of ASCs did not induce any serious adverse events related to ASC transplantation or any tumor development (even in nude mice) [38]. However, other studies have indicated that ASCs enhance tumorigenesis and metastasis of breast cancer cell lines and primary breast cancer samples [39,40]. Our recently published data showed that ASCs isolated from the subcutaneous abdominal adipose tissue of obese subjects have enhanced invasion toward breast cancer cells [41]. Prantl *et al*. demonstrated that ASCs promote prostate tumor growth [42]. Therefore, before use in human subjects, the safety of ASCs should be fully investigated and evaluated, particularly in patients with breast cancer and prostate cancer.

## Conclusions

This study suggests that the transplantation of human or mouse ASCs by oropharyngeal aspiration effectively attenuates lung injury and inflammation in a mouse model of LPS-induced ALI. These therapeutic effects may be partially due to the increased production of IL-10 in the lung following the cell-based treatment. A side-by-side comparison indicated that mASCs are more potent than hASCs in mediating the therapeutic effects following LPS-induced ALI in this mouse model. Therefore, adipose-derived stem cells may serve as a novel treatment modality for ALI, a medical challenge that currently lacks efficient therapies beyond supportive care.

## Abbreviations

ALI: acute lung injury; ARDS: acute respiratory distress syndrome; ASCs: adipose-derived stem cells; BAL: bronchoalveolar lavage; BALF: bronchoalveolar lavage fluid; BMSCs: bone marrow-derived mesenchymal stem cells; CCM: complete culture medium; CFU-F: colony-forming unit-fibroblasts; DMEM: Dulbecco's modified Eagle's medium; eGFP: enhanced green fluorescent protein; ELISA: enzyme-linked immunosorbent assay; GAPDH: glyceraldehyde-3-phosphate dehydrogenase; GM-CSF: granulocyte macrophage colony-stimulating factor; H&E: hematoxylin and eosin; hASCs: human adipose-derived stem cells; HBSS: Hank's balanced salt solution; IFN: interferon; IL: interleukin; IP: intraperitoneal; IV: intravenous; LPS: lipopolysaccharide; MIP: macrophage inflammatory protein; MSCs: mesenchymal stem cells; mASCs: mouse adipose-derived stem cells; MPO: myeloperoxidase; OA: oropharyngeal aspiration; PBS: phosphate-buffered saline; RT-PCR: reverse transcription-polymerase chain reaction; TNF-α: tumor necrosis factor alpha
